# First insights into chlorhexidine retention in the oral cavity after application of different regimens

**DOI:** 10.1007/s00784-021-03910-y

**Published:** 2021-04-06

**Authors:** Bashar Reda, Klaus Hollemeyer, Simone Trautmann, Dietrich A. Volmer, Matthias Hannig

**Affiliations:** 1 Clinic of Operative Dentistry, Periodontology and Preventive Dentistry, University Hospital, Saarland University, Building 73, 66421 Homburg, Saar Germany; 2 Physical Chemistry and Didactics of Chemistry, Saarland University, Campus B2 2, 66123 Saarbrücken, Germany; 3 Department of Chemistry, Bioanalytical Chemistry, Humboldt University of Berlin, Brook-Taylor-Strasse 2, 12489 Berlin, Germany

**Keywords:** Chlorhexidine, MALDI-TOF MS, Substantivity, Mouth rinse, Spray, Toothpaste

## Abstract

**Objectives:**

This in situ study aimed to determine and compare the chlorhexidine (CHX) retention in the oral cavity after the application of different CHX pharmaceutical regimens.

**Methods:**

Five volunteers used different CHX treatment regimens including mouth rinses, dental spray and toothpaste gel. After the application of the different CHX regimens, 2-μl samples were taken from saliva and buccal mucosa pellicle as well as the dental pellicle samples formed on standardized enamel surfaces. Sample collection was conducted at six time points within 12 h. Retention of CHX was measured using matrix-assisted laser desorption/ionization time-of-flight (MALDI-TOF) mass spectrometry.

**Results:**

CHX retention values in the oral mucosa pellicle were significantly higher than those in saliva. CHX remained in the mucosal pellicle at microgrammes per millilitre levels for 12 h after mouth rinsing, 10 h after spray application and 2 h after using the toothpaste. CHX was detected in the dental pellicle for at least 12 h after application of mouth rinsing and spray. Retention of CHX after mouth rinsing or spray application was significantly higher than the retention after using toothpaste.

**Conclusions:**

Oral mucosa was the favourable site for CHX retention. Higher mouth rinse concentration and longer rinsing time produced a slight increase in CHX retention. CHX spray provided considerable retention values, whereas toothpaste gel delivered the lowest retention after application. MALDI-TOF was a sensitive method with excellent limits of quantification for CHX detection.

## Introduction

Chemotherapeutic agents have the potential to inhibit plaque growth, reduce gingivitis and improve oral health in combination with mechanical plaque control [1].

Chlorhexidine (CHX), a bicationic bisbiguanide, is considered the most effective antimicrobial agent for chemical control of plaque formation and prevention of caries for more than 50 years [2–4]. In 1954, CHX was first introduced in the UK as a disinfectant and topical antiseptic [5]. CHX exhibits a wide spectrum of antibacterial activity that targets both Gram-positive and Gram-negative bacteria as well as yeast, dermatophytes and some lipophilic viruses [6]. The first clinical study demonstrating the ability of CHX to inhibit the formation and development of bacterial plaque was published in 1970 [2]. Since then, CHX has been delivered in a variety of formulations and vehicles, such as mouth rinse, spray, toothpaste, gel, varnish and slow-release devices. Solutions with alcohol or alcohol-free 0.2% CHX have been recommended as a mouth rinse in 10-ml volumes (20 mg dose). CHX solutions with a concentration of 0.12% are also used as a mouth rinse but in 15-ml volumes (18 mg dose) [7, 8]. Small doses of approximately 1.5 ml CHX (3 mg dose) delivered from a spray to tooth surfaces, particularly in physically and mentally handicapped groups, also showed good anti-biofilm activity [9–12]. Several systematic reviews concluded that tooth brushing with CHX-containing toothpaste is effective in controlling plaque and gingivitis to some degree, especially after using high concentrations of CHX. However, CHX toothpaste was not as effective as CHX mouth rinse [13, 14].

After application, CHX binds to many sites in the oral cavity and is slowly released to provide a sustained antimicrobial effect. This CHX considerable substantivity restricts bacterial proliferation for at least 24 h [15–18]. Unfortunately, this positive outcome of CHX is accompanied by side effects such as brown staining of teeth and restorations, unpleasant taste and an increase in calculus deposition [19, 20].

Previously, direct ultraviolet spectroscopy was often used for determining CHX concentrations; however, it is unspecific for CHX detection because of the distribution by several salivary components [21]. Therefore, techniques based on radiolabelling with carbon-14 [15] or fluorometric methods [22] were subsequently used. Due to the higher sensitivity, ^14^C-labelled CHX could be detected in saliva 24 h after administration, but this method is ethically inapplicable to humans. Fluorometric methods were limited to determine CHX in aqueous solutions and centrifuged saliva, but did not work in whole saliva samples. Different approaches of high-performance liquid chromatography (HPLC) have been used for CHX detection [23–26]. Additionally, solid-phase microextraction (SPME) was used to monitor free and total concentrations of CHX in pharmacokinetic investigations [27]. However, HPLC and SPME methods require generally long analysis times for multiple extraction and cleaning steps [28].

Matrix-assisted laser desorption/ionization time-of-flight (MALDI-TOF) mass spectrometry (MS) was used to detect CHX in microtome slices of a bacterially contaminated wound model treated with CHX [29]. Recently, we were able to determine and compare the CHX retention in samples from different oral locations by using MALDI-TOF [18]. The MALDI-TOF approach offers several advantages such as high sample-throughput, accurate mass analysis and identification and limited sample clean-up steps [30].

The present study aimed to use the aforementioned advantages of the MALDI-TOF technique to evaluate and compare the retention of CHX in the oral cavity after using different CHX delivery systems (rinse, spray, toothpaste gel) and treatment regimens, with the aim of discovering additional information on the pharmacokinetics and the efficiency of these different regimens at different oral sites.

## Materials and methods

### Human subjects

Oral cavity samples were collected for the present experimental study from five non-smoking volunteer subjects (3 females, 2 males), aged 24–36 years.

Visual oral examinations were carried out by an experienced dentist. The parameters used for this oral examination were PSR index (Periodontal Screening and Recording) for the determination of periodontal diseases [31] and ICDAS index (International Caries Detection and Assessment System) for the detection of active dental caries [32]. To avoid oral diseases that could potentially affect the oral fluid composition and play an unintended role in the CHX substantivity, all recruited subjects had an oral situation of Code 0 in both indices, PSR and ICDAS. This means that the subjects did not have any periodontal diseases and/or any evidence of dental caries. Additional exclusion criteria were conducted in the present study including the following: pregnancy, nursing women, antibiotic therapy within the previous three months, smoking and any systemic disease.

The number of volunteers included in the study was decided based on previous reports [15, 18, 21–27], where similar or even less numbers of subjects were included. All the volunteers who participated in the present study were chosen from the laboratory staff according to the aforementioned inclusion criteria. The subjects gave their informed written consent to participate in this study. The study protocols and informed consent were performed under the guidelines of the Declaration of Helsinki. Oral sample collection protocols were approved by the medical ethics committee of the Medical Association of Saarland, Germany (238/03, 2016).

### Study design

Experiments were performed over a period of 12 h and began at 7 am with the selected individual. Extensive oral hygienic procedure was performed at least 60 min before starting the experiment. No further oral hygienic procedures were performed until the end of the 12 h period of the experiment. The volunteers were instructed to take only one main food meal 5 h after the start of the experiment and they were allowed to consume different beverages during the time of the experiment.

The study lasted for about 1 year, starting with the conception and study design, then samples collection and CHX quantification, ending with data acquisition, statistical analysis and data interpretation.

### Reagents

CHX digluconate solutions with concentrations of 0.2% and 0.12% in 7% ethanol (Saarland University Pharmacy, Homburg, Germany), Chlorhexamed® Forte 0.2% alcohol-free spray (GlaxoSmithKline Consumer Healthcare GmbH & Co. KG, Bühl, Germany), Curasept ADS® 712 gel toothpaste with 0.12% CHX digluconate (Curaden Swiss GmbH, Stutensee, Germany) and GUM® Paroex® dental gel with 0.12% CHX digluconate (Sunstar GmbH, Schönau, Germany) were used for the different CHX regimens. Sterile water (B. Braun, Melsungen AG, Germany), acetonitrile 100% and trifluoroacetic acid 99% (VWR International GmbH, Darmstadt, Germany), as well as α-cyano-4-hydroxycinnamic acid (CHCA, Sigma-Aldrich, Steinheim, Germany), were used for matrix preparation. Disposable plastic spatulas (Ecospatula, Carl Roth GmbH & Co. KG, Karlsruhe, Germany) were used for scraping the buccal mucosa surface to collect the mucosal pellicle samples. MALDI 384 well stainless steel plates (Sciex, Darmstadt, Germany) were used as targets.

### Chlorhexidine regimens

Ten millilitres of 0.2% or 0.12% CHX digluconate mouth rinse was used for 30 and 60 s, respectively. Twelve squirts (one squirt equals 137 μl) of CHX digluconate spray were directly applied to the teeth (6 squirts at each jaw) and then rinsed with total squirts (1.644 ml of 0.2% CHX) for 1 min (the CHX digluconate dose after spray application was 3.288 mg). One gram of Curasept ADS® 712 gel toothpaste and 1 g of GUM® Paroex® gel toothpaste (both containing 1.2 mg of CHX digluconate) were used for 2-min brushing. The volunteers used the aforementioned CHX formulations (rinses, spray and toothpastes) according to the manufacturer’s instructions and there was no new treatment regimen investigated in the present study.

### Sample collection

**Fig. 1:**
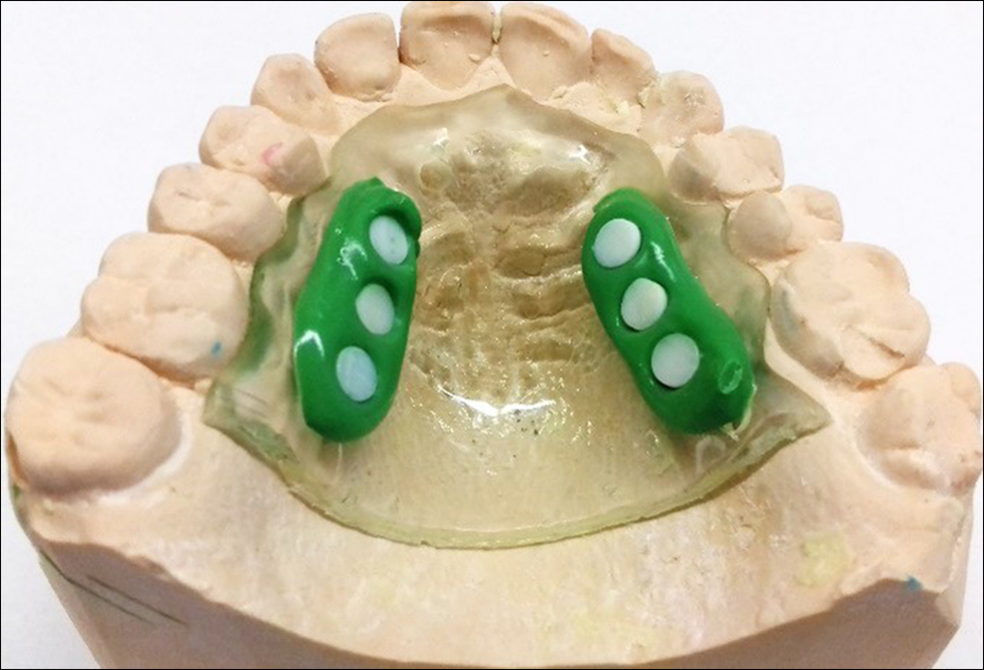
Palatal acrylic splint with mounted specimens to form the dental pellicle samples

Experimental samples were taken in the present study as follows: (1) 2 μl of sample was taken from the whole saliva; (2) 2 μl of sample of the buccal mucosa pellicle was taken from the collected pellicle after scraping the mucosal area with a plastic spatula; (3) dental pellicle sample was formed on one round enamel specimen with a standard surface area of 11.35 mm^2^. Enamel specimens were prepared from the labial surfaces of cattle incisor teeth. The specimens were polished by wet grinding with abrasive papers (up to 4000 grit) and fixed on an individual palatal splint (Fig. 1) before the exposure to the oral cavity. For the production of the palatal splint, alginate impression (Blueprint cremix®, Dentsply DeTrey, Konstanz, Germany) was made for the upper jaw of each subject to produce an elastic mould. Using this mould, a hard plaster model was made. A transparent custom-made acrylic splint (Thermoforming foils®, Erkodent, Pfalzgrafenweiler, Germany) covering the palatal area was made on the plaster model as a carrier of the enamel specimens. In order to better stabilize the mounted specimens, the splint was provided with small perforations. The enamel specimens were fixed to the individual acrylic splint using silicon impression material (president light body®, Colténe, Altstaetten, Switzerland).

After application of different CHX digluconate regimens, samples from each subject were taken at six time intervals (2 h, 4 h, 6 h, 8 h, 10 h, and 12 h). After removal from the oral cavity, samples were individually dissolved in 100 μl CHCA matrix solutions (10 mg/ml α-cyano-4-hydroxycinnamic acid, 70% acetonitrile and 0.07% trifluoroacetic acid) followed by a further dilution step to adjust CHX concentrations to the MALDI-TOF method. From each diluted sample, 5 spots of 0.5 μl each were pipetted via the dried-droplet method onto a polished MALDI target plate and allowed to dry [18].

### Calibration curve of chlorhexidine

A working solution of 0.1 mg/ml CHX in 7% ethanol was prepared by filling up 88.81 μl of stock solution of 0.2% of CHX digluconate in 7% ethanol to 1000 μl with matrix solution. This solution was further diluted twofold with matrix solution to 1.9×10^−7^ mg/ml. Five replicates of 0.5 μl each were taken from each aliquot and spotted onto the MALDI target plate.

### Recovery rate

Five working samples of CHX were prepared from the 0.2% and 0.12% CHX rinsing solutions, 0.2% CHX spray and 0.12% CHX toothpastes. These samples with CHX concentrations of 0.28 μg/ml, 0.17 μg/ml, 0.28 μg/ml, 0.17 μg/ml, and 0.17 μg/ml were obtained by diluting the original CHX products with CHCA matrix solution. Five replicate samples of 0.5 μl of each sample were spotted onto the MALDI target plate for measurement.

### MALDI-TOF mass spectrometry

MALDI-TOF analyses were performed on a Sciex (Darmstadt, Germany) 4800 TOF/TOF mass spectrometer in positive ion reflector mode over a range of m/z 370–600. The system uses a pulsed 200-Hz solid-state Nd:YAG laser with a wavelength of 355 nm. Laser energy was set to 2300 units for standards and samples. Source 1 voltage was set to 20 kV with a grid voltage of 16 kV. The reflector detector voltage was set to 2.19 kV. CHX standard (protonated monoisotopic mass [M_*i*_+H^+^]^+^ at m/z 505.21, monoisotopic decay product [M_*i*_+H^+^-NH_3_]^+^ at m/z 488.21 and the monoisotopic CHCA matrix dimer [2M_*i*_+H]^+^ at m/z 379.09 were used for internal mass calibration with a delay time of 600 ns. One average mass spectrum was obtained from 20 sub-spectra per spot using 25 accepted laser shots each. Mass tolerance was set to ± 0.1 u with maximum outlier of 50 ppm. Accepted calibration settings were used to measure sample spectra with a minimum signal to noise range of 20 and resolution (FWHM) >8000. The measured monoisotopic peaks were extracted into Microsoft Excel worksheets. Their absolute intensities (*a*_*i*_) were normalized by calculating the ratio of *I*_abs,ai_ and sum of absolute intensities of all compounds in the investigated m/z range according to *I*_rel,ai_= *a*_*i*_ /Σ (*a*). This resulted in relative intensities for the monoisotopic m/z 505.21 (=*I*_rel 505,ai_) of CHX. For each sample, 5 replicate measurements were performed using different sample spots and their relative intensities were averaged, resulting in a list of averaged monoisotopic mass intensities *I*_rel 505,ai_ for CHX. The averaged relative intensities were used for calculating CHX concentrations through the calibration curve.

### Statistical analysis

All CHX values were expressed as mean ± standard deviation and are presented in Tables 1, 2 and 3. The data were analysed with GraphPad Prism 8 (GraphPad Software, San Diego, CA, USA) using two-way repeated-measures analysis of variance (ANOVA) as two factors were changing during the experiment: sampling time and CHX regimen. Multiple comparisons were conducted with the Tukey test to reveal any significant differences in the oral CHX retention between the different CHX regimens. Additionally, two-way ANOVA was used to compare the retention of CHX in saliva and in the buccal mucosa pellicle. In this case, the two factors were the sampling time and the oral location. Again, multiple comparisons were conducted with the Tukey test to uncover potential significant differences in oral CHX retention between the different oral locations. Kruskal-Wallis test followed by Dunn’s multiple comparison test was performed to investigate the inter-individual variation in CHX retention after each CHX regimen at different oral sites. For all comparisons, statistical significance was defined as *p* < 0.05.

**Table 1:** Mean (±SD) concentrations (μg/ml) of chlorhexidine (CHX) in the buccal mucosal pellicle over 12 h after application of different CHX regimens

Chlorhexidine in the buccal mucosal pellicle (μg/ml)							
Time (h)	0.2% CHX - 60 s rinse^§^	0.2% CHX - 30 s rinse^§^	0.12% CHX - 60 s rinse^§^	0.12% CHX - 30 s rinse^§^	0.2% CHX spray^†^	0.12% CHX Curasept toothpaste	0.12% CHX Paroex toothpaste
2	284 ± 65.1	262.3 ± 78	232.9 ± 9.2	231.7 ± 25.7	156 ± 39.5	1.8 ± 1.9	2.7 ± 2.3
4	172 ± 51.4	133 ± 42.3	127 ± 64.5	124.7 ± 63.6	46.3 ± 22.2	0.9 ± 0.8	0.3 ± 0.2
6	36.2 ± 23.3	24.7 ± 15.9	19.9 ± 13.1	20.7 ± 13.1	13.8 ± 20.3	-	-
8	33.9 ± 18.8	11.6 ± 9.1	9.7 ± 5.1	7.5 ± 6.3	3.7 ± 2.4	-	-
10	20.9 ± 16.3	7.2 ± 7.3	3.6 ± 3.3	4.6 ± 2.8	2.2 ± 1.9	-	-
12	7.3 ± 5.2	2.1 ± 1.8	1.6 ± 3.3	1.3 ± 1.8	0.4 ± 0.4	-	-

- chlorhexidine less than the limit of quantification

^§^The differences in CHX retention between the rinsing regimens were not statistically significant (*p* > 0.05), except between 0.2% CHX rinsing for 60 s and 0.12% CHX rinsing for 60 s (*p* = 0.015), as well as between 0.2% CHX rinsing for 60 s and 0.12% CHX rinsing for 30 s (*p* = 0.005)

^†^The differences in CHX retention between spray application and all other CHX rinsing regimens were statistically significant (*p* < 0.05)

The differences in CHX retention between toothpaste application and all other CHX regimens were statistically significant (*p* < 0.05)

**Table 2:** Mean (±SD) concentrations (μg/ml) of chlorhexidine (CHX) in the saliva over 12 h after application of different chlorhexidine regimens

Chlorhexidine in saliva (μg/ml)							
Time (h)	0.2% CHX - 60 s rinse^§^	0.2% CHX - 30 s rinse^§^	0.12% CHX - 60 s rinse^§^	0.12% CHX - 30 s rinse^§^	0.2%CHX spray^†^	0.12% CHX Curasept toothpaste	0.12% CHX Paroex toothpaste
2	17.3 ± 10.7	25.7 ± 17.7	17.2 ± 6.8	11.4 ± 5.5	7.5 ± 5.6	0.1 ± 0.1	0.1 ± 0.1
4	10.7 ± 6.8	6.8 ± 4.7	8.9 ± 8.5	5.6 ± 3.9	3.3 ± 3.9	-	-
6	4.6 ± 4.2	1.9 ± 1.6	3.4 ± 3.9	0.6 ± 0.3	0.6 ± 0.5	-	-
8	2.3 ± 2.8	1.3 ± 1.2	1.4 ± 1.5	0.47 ± 0.3	0.4 ± 0.3	-	-
10	1.4 ± 1.2	0.5 ± 0.6	0.4 ± 0.2	0.07 ± 0.03	0.1 ± 0.2	-	-
12	0.5 ± 0.6	0.2 ± 0.1	0.2 ± 0.2	0.02 ± 0.03	-	-	-

- chlorhexidine less than the limit of quantification

^§^The differences in CHX retention between the rinsing regimens were not statistically significant (*p* > 0.05)

^†^The differences in CHX retention between spray application and all other CHX rinsing regimens were not statistically significant (*p* > 0.05), except with 0.2% CHX rinsing for 60 s (*p* = 0.03)

The differences in CHX retention between toothpaste application and all other CHX regimens were statistically significant (*p* < 0.05)

**Table 3:** Mean (±SD) retention (μg/cm^2^) of chlorhexidine (CHX) in the dental pellicle over 12 h after application of different chlorhexidine regimens

Chlorhexidine in the dental pellicle (μg/cm^2^)							
Time (h)	0.2% CHX - 60 s rinse^§^	0.2% CHX - 30 s rinse^§^	0.12% CHX - 60 s rinse^§^	0.12% CHX - 30 s rinse^§^	0.2%CHX spray^†^	0.12% CHX Curasept toothpaste	0.12% CHX Paroex toothpaste
2	0.33 ± 0.15	0.25 ± 0.09	0.32 ± 0.25	0.26 ± 0.15	0.15 ± 0.11	0.02 ± 0.02	0.01 ± 0.01
4	0.28 ± 0.09	0.18 ± 0.08	0.22 ± 0.17	0.18 ± 0.11	0.07 ± 0.04	0.01 ± 0.01	0.01 ± 0.01
6	0.21 ± 0.15	0.13 ± 0.06	0.09 ± 0.03	0.09 ± 0.08	0.05 ± 0.04	-	-
8	0.12 ± 0.06	0.10 ± 0.04	0.05 ± 0.04	0.05 ± 0.03	0.04 ± 0.04	-	-
10	0.06 ± 0.02	0.05 ± 0.04	0.03 ± 0.02	0.04 ± 0.03	0.02 ± 0.02	-	-
12	0.06 ± 0.01	0.04 ± 0.03	0.02 ± 0.01	0.02 ± 0.02	0.02 ± 0.02	-	-

- chlorhexidine less than the limit of quantification

^§^The differences in CHX retention between the rinsing regimens were not statistically significant (*p* > 0.05)

^†^The differences in CHX retention between spray application and all other CHX rinsing regimens were not statistically significant (p > 0.05), except with 0.2% CHX rinsing for 60 s (*p* = 0.005)

The differences in CHX retention between toothpaste application and all other CHX regimens were statistically significant (*p* < 0.05)

It is important to note that repeated-measures ANOVA test is quite sensitive to violations of the assumption of sphericity or circularity. This violation could happen when the repeated measurements are made too close together so that random factors that cause a particular value to be high (or low) do not wash away or dissipate before the next measurement. To avoid violating the assumption of sphericity in the present study, an important washout period of 10 days was applied to eliminate any rest of CHX in the oral cavity from a treatment regimen which can affect the retention values of the next treatment regimen. Additionally, the order of treatments was random and each subject followed a different sequence of CHX treatment regimens in his experimental trial.

## Results

### Chlorhexidine calibration curve and recovery rate

**Fig. 2:**
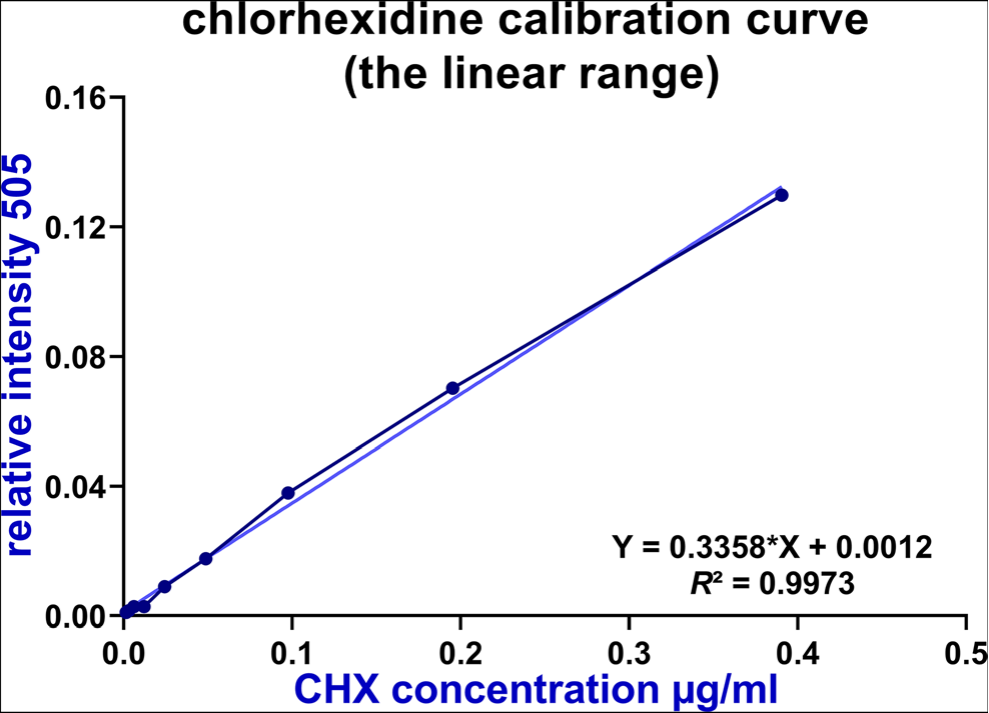
Calibration curve of chlorhexidine (CHX)-concentrations versus their relative intensity values. The linear range extends from 1.5×10^−3^ to 0.39 μg/ml. The linear equation and the quadratic correlation coefficients are given for this range

Mass accuracies for the [M_*i*_+H^+^]^+^ m/z 505.21 measurements were 25 ppm on average based on absolute m/z differences. The calibration curves were linear from 1.5×10^−3^ to 0.39 μg/ml of CHX free base with a regression correlation coefficient of *R*^2^ = 0.9972 (Fig. 2).

The lowest concentration of the calibration curve was set as the limit of quantification for CHX. Detection saturation was observed for concentrations >0.39 μg/ml; concentrations <1.5×10^−3^ μg/ml gave S/N ratios of 20 or less and prevented CHX quantification. After determining CHX concentration in the matrix sample mixtures, the dilution factor was applied for calculating the CHX concentrations in the original oral samples.

Additionally, the recovery was assessed by comparing the obtained free CHX base content from the MALDI-TOF analyses to the original sample concentrations of different CHX regimens. The recovery rates of CHX in the present study were between 95.03 and 98.86%.

### Oral concentrations of chlorhexidine after application of different regimens

**Fig. 3:**
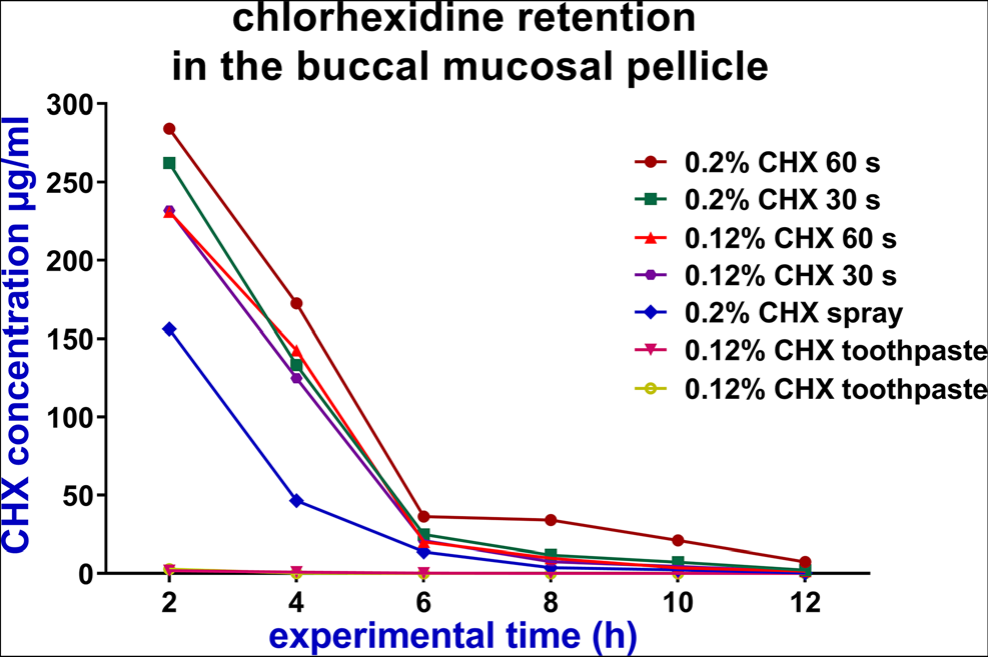
The mean concentrations (μg/ml) of chlorhexidine (CHX) in the buccal mucosal pellicle over 12 h after application of different chlorhexidine regimens

**Fig. 4:**
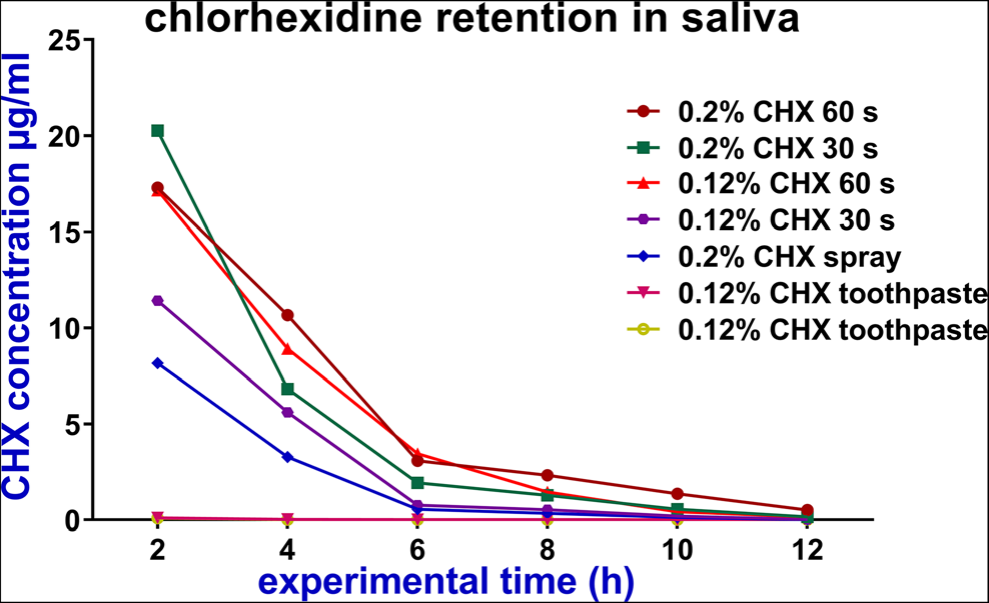
The mean concentrations (μg/ml) of chlorhexidine (CHX) in saliva over 12 h after application of different CHX regimens

**Fig. 5:**
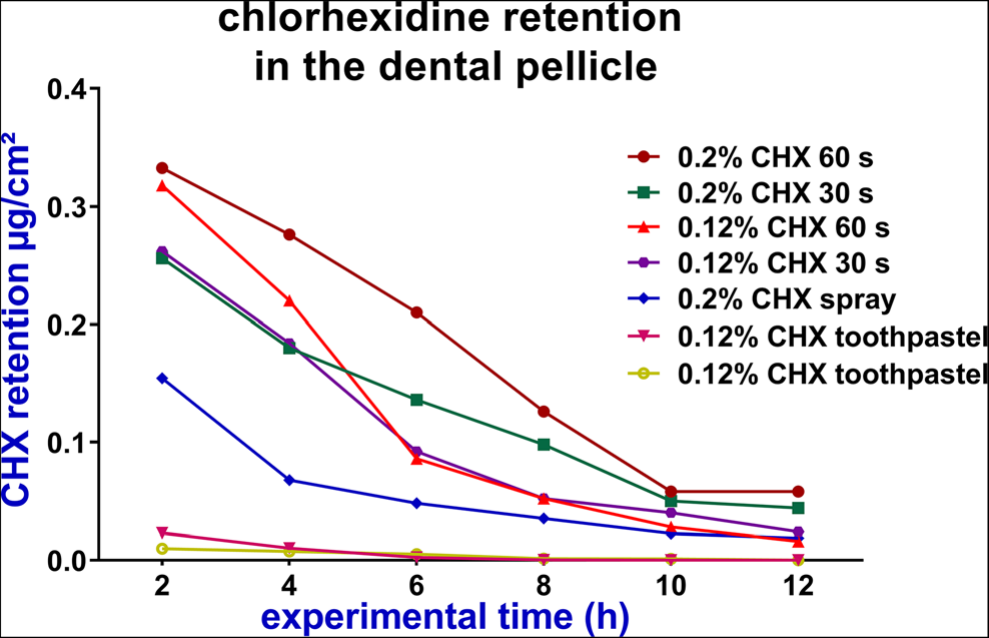
The mean retention (μg/cm^2^) of chlorhexidine (CHX) in the dental pellicle over 12 h after application of different CHX regimens

The oral CHX concentrations at different sites continuously decreased after application of different CHX regimens (Figs. 3, 4 and 5). The highest decline rates of CHX concentration in the oral cavity occurred during the first 6 h after application followed by a time period of at least 12 h with concentrations levels in the microgrammes per millilitre range (Tables 1 and 2).

After application of the different CHX regimens, the retention rates of CHX in the buccal mucosa pellicle were always higher compared to the retention in saliva (Tables 1 and 2).

After mouth rinsing, there were relatively higher retentions in saliva, mucosal pellicle and dental pellicle when the mouth rinse was performed either at a higher concentration of CHX or at longer rinsing time. The CHX retention in the buccal mucosa pellicle after the application of 0.2% CHX for 60 s regimen was significantly more than the retention after using 0.12% CHX for 60 s regimen or 0.12% CHX for 30 s regimen (*p* = 0.015, *p* = 0.005; respectively). However, the differences between the rinsing protocols in the saliva and the dental pellicle were not statistically significant (*p* > 0.05) (Tables 2 and 3).

Considerable retention was detected after CHX spray application. This retention lasted in the oral cavity for at least 12 h (Table 1). Furthermore, there were no significant differences regarding the CHX retention between the spray application and the rinsing regimens in saliva and dental pellicle except with 0.2% CHX rinsing for 60 s (*p* = 0.03, *p* = 0.005; respectively). However, in the buccal mucosa pellicle, the differences between the spray application and the four rinsing regimens were statistically significant.

In general, the retention of CHX changed as the dosage of the CHX in different regimens changed. When the dosage of the drug increased from 1.2 mg in the toothpaste to 3.28 mg in spray to 20 mg in the mouth rinse, the CHX concentrations in the mucosal pellicle also increased from 2.74 to 156.08 μg/ml and finally to 284.12 μg/ml, 2 h after application (Table 1). Similarly, as shown in Table 3, there was significantly lower adsorption of CHX on the dental pellicle when the toothpaste was used (0.01–0.03 μg/cm^2^) compared with the retention obtained when the different rinsing protocols were tested (0.25–0.6 μg/cm^2^) at 2-h time point.

Lastly, no significant inter-individual differences were detected after the statistical comparisons between the volunteers regarding the CHX retention after each investigated regimen in each oral location.

## Discussion

This oral pharmacokinetic study investigated CHX retention in different oral sites regarding different variables that may affect this retention in the oral cavity, such as variations of the rinsing time, drug concentration and delivery system.

MALDI-TOF was used for quantitative analysis of CHX, as the alternatives HPLC and SPME techniques are time-consuming and labour-intensive, despite their selectivity, sensitivity and accuracy [28]. The characteristic isotope patterns of the two chlorine isotopes (^35^Cl/^37^Cl) yielded three distinctive ions (100% [^35^Cl^35^Cl]; 60% [^35^Cl^37^Cl+^37^Cl^35^Cl]; 9% [^37^Cl^37^Cl]). Together with the accurate m/z determination, the chlorine isotope patterns allowed unequivocal identification of CHX in the measured mass spectra, as interfering endogenous physiological components do not contain chlorine. Neither salivary components, intakes of food and beverages nor MALDI matrix signals interfered with the CHX isotope patterns. The low quantification limit of 1.5×10^−3^ μg/ml and the extended linear dynamic range both readily demonstrated the fit-for-purpose of MALDI-TOF for quantifying CHX at very low concentrations, combined with high recovery rates of CHX (95–98%). These analytical figures of merit were comparable to previous studies using HPLC techniques, which exhibited recovery rates between 93.2 [23] and 97.8% [26], showing consistency throughout different studies and techniques.

During the analysis of samples from the buccal mucosa pellicle, dental pellicle and saliva, the CHX concentrations varied at different oral locations in and between each subject. Several previous studies showed the same inter-individual variation after CHX application [15, 18, 24–26]. It appears that these individual differences depended on individual oral environment and food habits during the experiment. For example, consuming acidic foods and drinks can lower the pH within the oral cavity leading to reduced CHX retention [26, 33]. However, the inter-individual variations in the present study were not statistically significant. Also, we investigated the intra-individual variation in a previous retention study [18], where experimental trials were replicated on three consecutive weeks. The intra-individual differences of CHX retention were not statistically significant in the aforementioned study.

The concentrations of CHX in the buccal mucosa pellicle remained higher than 100 μg/ml for 4 h and then decreased over time, mostly because of the main food meal intake. The current results showed the CHX retention in the buccal mucosa pellicle to be more than ten times higher than the CHX retention in the saliva at all-time points. This might be explained by the high affinity of CHX to the oral mucosa. Such an affinity was noticed in a previous in vitro study, which suggested that the presence of proteins in pellicles and buccal epithelial cell membranes promote the adsorption of CHX [26]. Similar results were also obtained in a previous in situ study by analysing samples from different oral sites including the mucosal pellicle and saliva [18], where CHX retention in the mucosal pellicle was significantly higher than in the saliva. However, the sampling techniques in both studies differed. In the previous study, samples were taken using micro-brushes, whereas in the present study 2 μl samples were taken from the buccal mucosal pellicle and the saliva.

The concentrations of salivary CHX remained at microgrammes per millilitre levels up to 8 h after mouth rinsing. These results are in good agreement with previous studies of salivary CHX retention [18, 25–27].

Raising the CHX concentration or prolonging the rinsing time lead to a slight increase in CHX retention in the mucosal pellicle, saliva and dental pellicle. The results of the present study suggest that the maximum level of CHX retention is achieved when the 0.2% CHX rinsing regimen is used for 60 s. Such retention results are in line with the conclusions of a systemic review [34], where the authors found that there was a small but significant difference in favour of the 0.2% CHX concentration with regards to biofilm inhibition effects. It was also reported in previously published studies that there was higher retention by rising the CHX concentration from 0.1 to 1 mg/ml [26]. Bonesvoll and co-workers raised CHX retention by raising the concentration or prolonging the rinsing time [33]. However, the authors noted that there was a slight deviation towards less retention at the highest concentration tested. Such a retention saturation could be due to the limited binding capacity for CHX in the oral surfaces, which would explain why there was only a small difference in the retention values between 0.2 and 0.12% in this study. Additionally, this finding indicates that increasing the concentrations of CHX for more than 0.2% or rinsing with more than 10 ml (more than 20 mg CHX dose) will not necessarily induce significantly higher CHX retention and more persistent bacteriostatic activity because of the binding capacity for CHX in the oral cavity.

Attempts to overcome the side effects of prolonged CHX use have resulted in the development of different CHX regimens with various delivery systems and vehicles. These CHX regimens employ smaller quantities of CHX and deliver the drug to specific sites in the oral cavity. The CHX spray is one of these regimens, which offers a simple and rapid method to use very small doses of CHX of approximately one-seventh of the dose from a rinse. The CHX spray offers an effective plaque-inhibiting activity [10, 35]. The spray approach is particularly useful for elderly people and physically and mentally handicapped groups [9, 11, 36, 37]. This approach also allows focusing on treatments of specific regions such as periodontal or implant surgical sites [38, 39]. The results of the present in situ study are in line with the conclusions obtained from the aforementioned clinical studies. It was shown in the current study that a considerable CHX retention is found after the spray application, which might provide adjunctive benefits to oral hygiene and gingival health with minimal side effects.

Adding CHX in dentifrice formulations to get both beneficial approaches, mechanical and chemical cleaning is thought to be helpful for patients with periodontal disease. However, some components in the toothpaste gel are sometimes anionic substances such as sodium lauryl sulphate (SLS). These substances could interfere with the biochemical pathway (bioavailability) of the CHX by forming salts of low solubility and low antibacterial activity [40]. Therefore, two different kinds of CHX toothpaste were tested in this study, one with SLS (Paroex) and another without it (Curasept), to determine the retention of CHX in the oral cavity and the effect of SLS on such retention.

The results of the present study showed no significant difference between toothpaste with or without SLS regarding retention of CHX in the oral cavity. Furthermore, the CHX from the toothpaste was retained in the oral cavity at low concentrations and not to the same extent after a CHX mouth rinse or even after a CHX spray application. The reason behind this low retention could be due to the mechanical action of the tooth brushing that can remove the newly attached CHX particles at the dental pellicle. Additionally, rinsing the mouth several times with water after tooth brushing might also wash away the CHX particles from the oral cavity surfaces. In contrast, most of the retained CHX after mouth rinsing or spray application is supposed to stay in the oral cavity after application because patients should not further rinse their mouths with water after using the mouthwash or spray. To overcome the problem of washing away retained CHX after tooth brushing, it was suggested in previous studies that CHX gel could be delivered in trays or applied directly to teeth surfaces with the finger [41]. This style of application, especially with high CHX concentration, provided more antibacterial efficiency and caused a significant reduction of plaque scores and gingival index [14]. Such an effectivity was comparable with the mouth rinse in some studies [41, 42].

Another factor that can influence the retention of CHX after toothpaste application is the dosage of the drug. It was shown in a systematic review that the positive effects of CHX toothpaste were noticeable only when the concentration of CHX was higher than 0.6% [13]. In this context, the 0.12% CHX toothpaste used in this study delivered a low dosage of only 1.2 mg to the oral cavity, which is much lower than a 20 mg CHX dose delivered by the rinsing. This small dosage of 1.2 mg CHX might explain why there was small retention after application and almost no substantivity after 2 h. It is also important to point out that CHX retention in the mucosal pellicle after toothpaste application was recorded at 2 μg/ml at the 2-h time point. This concentration may be sufficient as an antibacterial agent for primary cariogenic bacteria such as *Streptococcus mutans*, *Actinomycetes* and *Lactobacilli* [43]. However, it may be insufficient to destroy the subgingival bacteria, which requires minimum inhibitory concentrations (MICs) ranging from 8 to 250 μg/ml [44]. These MICs can only be obtained after mouth rinsing or spray application.

Lastly, considering the main limitation of this study which was the limited number of volunteers, further studies with a higher number of subjects will be very helpful to confirm the results of the present study and clarify more the effects of different CHX delivery systems and treatment regimens on the CHX retention in the oral cavity.

## Conclusions

The results of the present study suggest that CHX concentration and the CHX delivery system play an essential role in CHX retention in the oral cavity after application. The retention of CHX was much higher after mouth rinsing as compared to toothpaste application. In the current study, the application of 0.2% CHX for 60 s showed the best retention values, which can provide a direct bactericidal and persistent bacteriostatic activity in the oral cavity. This would be beneficial when a significant reduction of oral bacteria is required, such as for oral surgery preparations or for the treatment of periodontal diseases.

Additionally, according to the high retention values recorded after the spray application, this type of CHX application can be recommended, especially in special cases such as for physically and mentally handicapped populations.

The current study also suggests that oral surfaces are the main reservoirs of CHX. Subsequently, saliva, as a constantly cleared and renewed substance in the oral cavity, would acquire CHX at low concentrations from other oral locations such as the mucosal surfaces and dental pellicle. These low CHX concentrations in the saliva would maintain a persistent bacteriostatic effect against oral flora [16, 17].
